# Recent advances in modelling of cerebellar ataxia using induced pluripotent stem cells

**DOI:** 10.29245/2572.942x/2017/7.1134

**Published:** 2017-07-10

**Authors:** Maggie M. K. Wong, Lauren M. Watson, Esther B. E. Becker

**Affiliations:** 1 Department of Physiology, Anatomy and Genetics, University of Oxford, United Kingdom

**Keywords:** iPSC, Cerebellum, Ataxia, Disease modelling, Purkinje cells, Development, Neurodegeneration

## Abstract

The cerebellar ataxias are a group of incurable brain disorders that are caused primarily by the progressive dysfunction and degeneration of cerebellar Purkinje cells. The lack of reliable disease models for the heterogeneous ataxias has hindered the understanding of the underlying pathogenic mechanisms as well as the development of effective therapies for these devastating diseases. Recent advances in the field of induced pluripotent stem cell (iPSC) technology offer new possibilities to better understand and potentially reverse disease pathology. Given the neurodevelopmental phenotypes observed in several types of ataxias, iPSC-based models have the potential to provide significant insights into disease progression, as well as opportunities for the development of early intervention therapies. To date, however, very few studies have successfully used iPSC-derived cells to model cerebellar ataxias. In this review, we focus on recent breakthroughs in generating human iPSC-derived Purkinje cells. We also highlight the future challenges that will need to be addressed in order to fully exploit these models for the modelling of the molecular mechanisms underlying cerebellar ataxias and the development of effective therapeutics.

## Cerebellar ataxias

The cerebellar ataxias are a clinically and genetically diverse group of neurological disorders that primarily affect the cerebellum. Characterized by a progressive loss of motor coordination, other symptoms include dysarthria and disturbance of eye movements^1^. Cerebellar ataxias may be acquired or inherited, with hereditary ataxias classified into autosomal dominant, autosomal recessive, X-linked or mitochondrial forms. Over 60 genetic subtypes have been identified to date^2^–^4^. Among these, the autosomal recessive disorders Friedreich ataxia (FRDA) and ataxia-telangiectasia (A-T), as well as the autosomal dominant polyglutamine spinocerebellar ataxias (SCAs) are the most studied forms of ataxias. Despite significant clinical and genetic heterogeneity, emerging evidence points to the existence of common pathogenic mechanisms that may be shared by several genetically distinct forms of cerebellar ataxias (reviewed in^5^–^8^). However, it is still unclear how the proposed pathological pathways ultimately result in cerebellar dysfunction and degeneration, predominantly affecting Purkinje cells.

Understanding disease mechanisms is key to treating neurodegenerative disorders. The heterogeneous nature of the cerebellar ataxias combined with the unavailability of human brain tissue and the lack of reliable disease models have, however, hampered our understanding of the molecular disease mechanisms underlying cerebellar ataxias and thus, the development of effective therapies. Although mouse models of several cerebellar ataxias, including FRDA and SCAs, have provided valuable insights into the pathophysiology of these disorders (reviewed in^9^), many questions remain about the observed species differences in disease phenotypes and the effectiveness of potential drugs in clinical trials. To help translate research from animal models into novel treatments for ataxia patients, it is essential to validate findings in the relevant affected human cell types, particularly in cerebellar Purkinje cells. The current obstacles might be overcome by exploiting recently developed human induced pluripotent stem cell (iPSC) technology and neuronal differentiation protocols.

## Generation of cerebellar neurons using human iPSCs

Major advances in the field of stem cell technology have allowed for the robust reprogramming of human somatic cells into iPSCs^10^–^12^. Human ataxia patient-specific iPSCs, which can subsequently be differentiated into specific neuronal subtypes and other affected cell types in cerebellar ataxias, would be an ideal tool for studying the pathogenic mechanisms in human and disease-relevant neurons, and might also be highly valuable for high-throughput drug screening *in vitro*^13^,^14^. Cerebellar Purkinje cells are particularly vulnerable in cerebellar ataxia but difficult to differentiate *in vitro,* owing to their large size, complex morphology, unique firing properties and extensive period of maturation^8^,^13^. However, significant breakthroughs have been achieved recently and are reviewed below.

The generation of cerebellar neurons from human iPSCs aims to recapitulate the complex *in vivo* molecular events that direct the specification of cerebellar neurons, and circuit formation during human embryogenesis^15^–^17^. Early protocols attempted cerebellar differentiation using either mouse or human embryonic stem cells (ESCs)^18^–^21^. These approaches sought to mimic the *in vivo* signals involved in early cerebellar development by delivering inductive signals, including bone morphogenic proteins, mitogens and neurotrophins, in a step-wise fashion throughout the differentiation process. However, the resulting cerebellar cultures only contained a small proportion of Purkinje cells and a relatively high number of cerebellar granule cells^18^,^20^.

Muguruma *et al.* adopted a different approach, which aimed to induce endogenous signals mimicking cerebellar patterning, by adding a combination of only three factors (insulin, fibroblast growth factor 2 (Fgf2) and cyclopamine) to floating aggregates of mouse or human ESCs^22^,^23^. Followed by dissociation, fluorescence-activated cell sorting for KIRREL2/NEPH3-positive Purkinje cell progenitors, and subsequent co-culture with mouse primary rhombic lip-derived granule cell precursors, this method resulted in the generation of Purkinje cells at a much higher efficiency than had previously been achieved. The differentiated cells expressed mature PC markers including L7/PCP2 and GRID2, and possessed a characteristic Purkinje cell morphology with an extensively arborized dendritic tree. Moreover, these cells displayed the distinctive firing pattern of Purkinje cells, suggesting that they were functionally mature^22^,^23^.

Wang *et al.* used a similar self-inductive approach to produce Purkinje cells from human iPSCs co-cultured with human foetal cerebellar slices^24^. Although these cells appeared to display Purkinje cell-specific markers and were electrically active, they did not possess the characteristic morphology of mature Purkinje cells. Moreover, this protocol is likely to be limited in its uptake due to associated ethical issues.

Most recently, the Mugumura group used a very similar approach to their original method to generate Purkinje cells from iPSCs, but with the inclusion of two additional growth factors (brain-derived neurotrophic factor (BDNF) and neurotrophin-3 (NT-3)) in their maturation medium^25^. After co-culture with mouse primary granule cell precursors for 75 days, the human iPSC-derived neurons possessed extensively branched dendritic trees with a thick stem, characteristic of mature Purkinje cells, and expressed the mature PC-specific markers L7/PCP2 throughout soma and dendrites, and the synaptic receptor GRID2 on dendritic spines. Thus, this protocol not only offers a shorter and more feasible protocol to generate Purkinje cells, but also produces more mature looking PCs that more closely resemble those *in vivo.*

Despite this significant progress, a number of challenges remain to be addressed before researchers can fully exploit iPSC-based cerebellar models. Dissociated cultures of neurons allow for close monitoring of their individual development throughout differentiation; however, their maturation and survival is limited compared to neurons developing in the embryonic brain, likely due to the lack of appropriate support in *in vitro* cultures^26^,^27^. Moreover, disease phenotypes affecting synapse formation, dendritic retraction and neuronal migration^28^,^29^, which may only arise from interactions between cells and their environment, may be lost in these two-dimensional cultures.

A way forward to overcome this limitation might be the use of organoids, self-organizing *in vitro* structures that include multiple cell types and mimic the *in vivo* architecture of an organ^30^. Despite the relative success in generating organoids mimicking development of the cerebral cortex (reviewed in^30^,^31^), only one study has thus far reported the generation of cerebellar organoids. Muguruma *et al.* showed that the addition of Fgf19 and stromal cell-derived factor 1 (Sdf1) during differentiation promoted the self-organisation of human ESCs into polarized three-layer structures reminiscent of the human embryonic cerebellum^23^. To date, however, no studies have reported the generation of cerebellar organoids from human iPSCs. Moreover, it remains a question whether self-induction alone is sufficient for generating a functionally mature human cerebellum *in vitro*. While long-term culture is needed for the maturation of multiple cell types that form active neuronal networks in spatially organized manner, the lack of mechanical support, as well as nutrient and gas exchange, cause cell death within organoids and therefore limit culture duration and maturity^31^. To overcome this, cerebellar organoids could be grown in spinning bioreactors to prolong their viability by improving nutrient and gas exchange^32^. Alternatively, the self-induction approach could be used to generate progenitors of various cerebellar subtypes, which could subsequently be plated onto biodegradable scaffolds to support their organization into a layered three-dimensional structure^30^. In such a system, microenvironmental niches surrounding progenitors have been shown to promote terminal differentiation and neuronal circuit formation^28^.

Another limitation of the studies so far is the lack of information on the relative maturity of the generated Purkinje cells and their resemblance to adult human Purkinje cells *in vivo*. Indeed, transcriptomic analysis of other iPSC-derived neuronal models has revealed that the generated neurons are still in an embryonic state, despite the fact that individual neurons have been stained for mature markers (reviewed in^29^). Such immature cells may be beneficial for the study of developmental aspects of disease. However, fully mature and functional Purkinje cells will be needed to study the cerebellar degeneration in ataxias due to the late disease onset in patients.

Lastly, the reproducibility of the developed protocols remains a challenge. Although revolutionary in the field, none of the abovementioned studies have been reproduced by other research groups, likely due to their complexity and long differentiation times. Moreover, substantial differences between batches of iPSC differentiation experiments remain a general issue in the field^27^. To allow for the extensive applications of iPSC-based models, therefore, it is essential to explore ways to shorten the differentiation process, facilitate the maturation of cerebellar neurons and reduce the variability of the protocols. Future studies should focus on detailed electrophysiology, as well as transcriptomic, proteomic and metabolomic analyses of iPSC-derived Purkinje cells. Moreover, it will be vital to compare the characteristics of the generated cerebellar cells to available data from human embryonic and adult cerebellum to stage the iPSC-derived models appropriately.

## Disease modelling of cerebellar ataxias

The iPSC-based cerebellar cultures described offer pioneering opportunities to study and model the disease processes underlying cerebellar ataxias in affected human neuronal cell types. Interestingly, mounting evidence from cell and animal models indicates that abnormal Purkinje cell development, including impaired dendritic arborisation, spine development and synaptogenesis, and related functional changes, may contribute to the pathogenesis of ataxias, including neurodegenerative ataxias^15^. Thus, it will be extremely interesting to investigate whether iPSC-derived human cerebellar neurons display any developmental abnormalities. In addition, these cultures will allow us, for the first time, to unravel the processes that might underlie the cell-specific vulnerability of human cerebellar Purkinje cells.

To date, fewer than 20 studies have reported investigations into cerebellar ataxia, including FRDA, A-T and polyglutamine SCAs, using human iPSCs. The majority of these studies have been performed in neurons without regional specification, as well as in other affected cell types (reviewed in^13^,^14^). None of these studies have yet successfully recapitulated the specific cerebellar neuronal dysfunction and degeneration known to characterize these conditions.

In human iPSC-based studies of FRDA, which is caused by an intronic repeat expansion in the *FXN* gene encoding Frataxin, disease-relevant phenotypes such as reduced Frataxin mRNA and protein levels as well as mitochondrial defects were observed in cardiomyocytes and peripheral sensory neurons, two of the affected cell types in FRDA^33^–^36^. In the case of A-T, chromosomal instability and cell cycle checkpoint defects, resulting from mutations in the *ATM* gene encoding ataxia-telangiectasia mutated (ATM) protein, have been found to reduce the efficiency of the reprogramming of A-T iPSCs^37^. Although A-T iPSCs can be induced to differentiate into neuronal cells^37^, it remains to be seen whether they are able to generate Purkinje cells.

A handful of studies have focused on the characterization of iPSCs or iPSC-derived neurons from patients with polyglutamine SCAs, including SCA2, 3, 6 and 7^25^,^38^–^41^. To date, only one study has examined the disease phenotype in the context of the affected neuronal cell type. Ishida *et al.* recently reported the differentiation of iPSCs into Purkinje cells from SCA6 patients that harbour a polyglutamine expansion in the calcium channel Cav2.1^25^. Patient Purkinje cells were more vulnerable to depletion of thyroid hormone triiodothyronine (T3), an important growth hormone for maturation and maintenance of Purkinje cells, as demonstrated by a decrease in cell number and reduction in dendritic arbours in patient cells compared to controls^15^,^42^. This study highlights the potential of patient iPSC-derived Purkinje cells to model developmental and disease-relevant ataxia phenotypes. Of note, the SCA6 Purkinje cells only showed abnormal dendritic development upon stress but not under normal culture conditions, suggesting that environmental triggers might be crucial to causing disease phenotypes. It will be interesting to see whether SCA6 phenotypes that have been observed in human post-mortem brain material as well as in SCA6 animal models, including calcium overload, cytoplasmic aggregation of expanded Cav2.1 protein, nuclear localization of the cleaved Cav2.1 C-terminus and the resulting perturbation of gene expression^5^,^43^, can be recapitulated in the iPSC-derived SCA6 model.

Notably, the observed developmental phenotype in the SCA6 Purkinje cells could be rescued using two compounds, thyroid releasing hormone and riluzole, demonstrating the potential of patient-specific iPSC-derived cerebellar neurons to be used for drug screening and personalized therapy.

## Conclusion

Recent breakthroughs in generating human iPSC-derived cerebellar neurons offer unprecedented opportunities to study the disease mechanisms underlying cerebellar ataxias. This exciting methodology will be particularly powerful when combined with other cutting-edge technologies including genome engineering, single-cell sequencing and high-resolution microscopy. Together, iPSC-derived cerebellar models are poised to advance our understanding of the disease mechanisms underlying the cerebellar ataxias, thereby enabling the development of more effective treatment options for patients in the future.

## Figures and Tables

**Figure 1:**
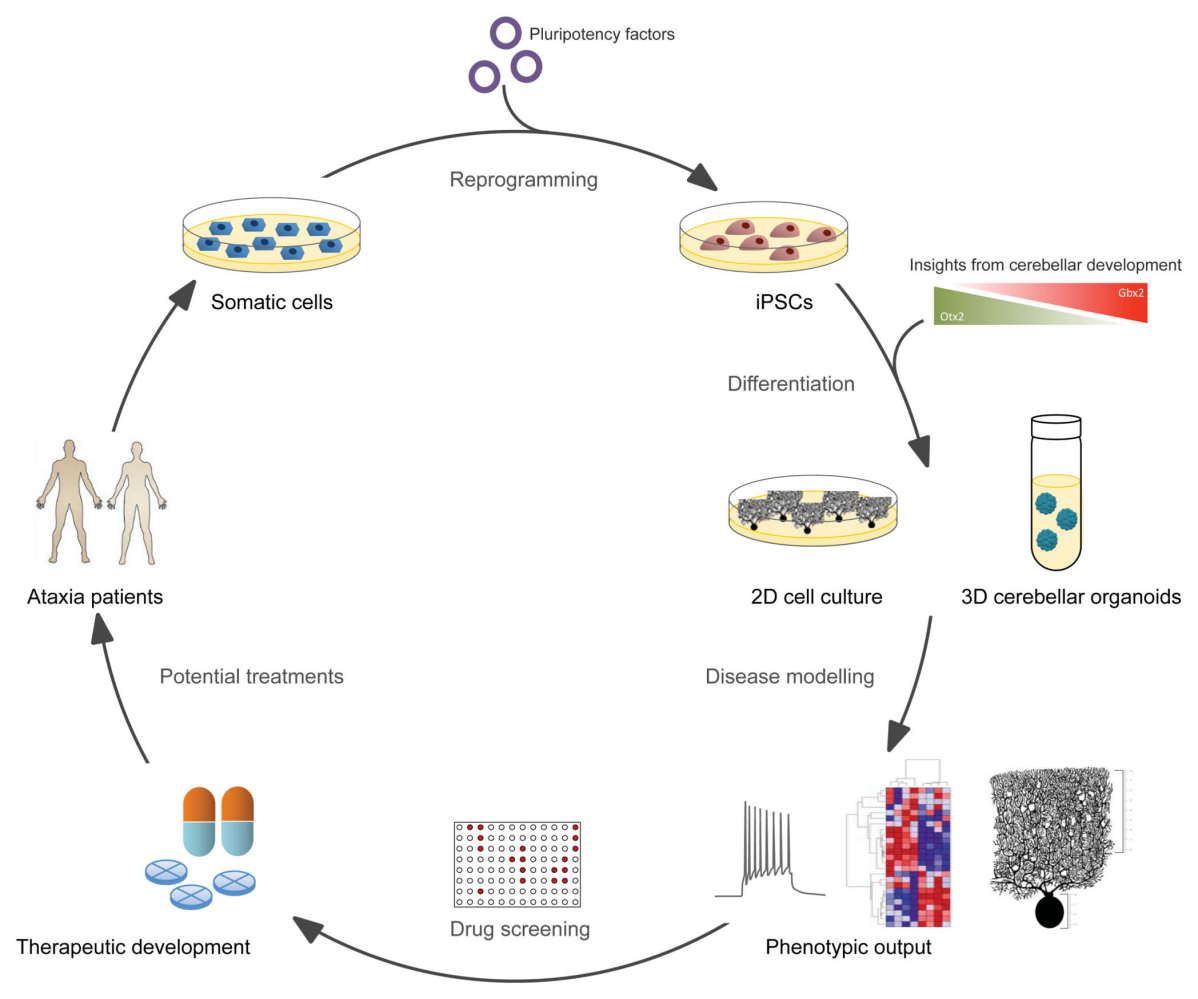
Schematic overview of iPSC-based disease modelling for cerebellar ataxias. Somatic cells from patients are reprogrammed into iPSCs by the introduction of pluripotency factors. These iPSCs can then be differentiated into disease-relevant cell types in either 2D- or 3D-format, following protocols that mimic cerebellar developmental cues *in vitro*. Cell culture models may then be used as a platform for the investigation of disease phenotypes, as well as for drug screening, with the ultimate goal of delivering effective therapies to patients.
